# Machine-Learning-Aided Understanding of Protein Adsorption on Zwitterionic Polymer Brushes

**DOI:** 10.1021/acsami.4c01401

**Published:** 2024-05-03

**Authors:** Hiroto Okuyama, Yuuki Sugawara, Takeo Yamaguchi

**Affiliations:** Laboratory for Chemistry and Life Science, Tokyo Institute of Technology, Yokohama 226-8501, Japan

**Keywords:** antifouling, zwitterionic polymer, protein adsorption, machine learning, polymer brush

## Abstract

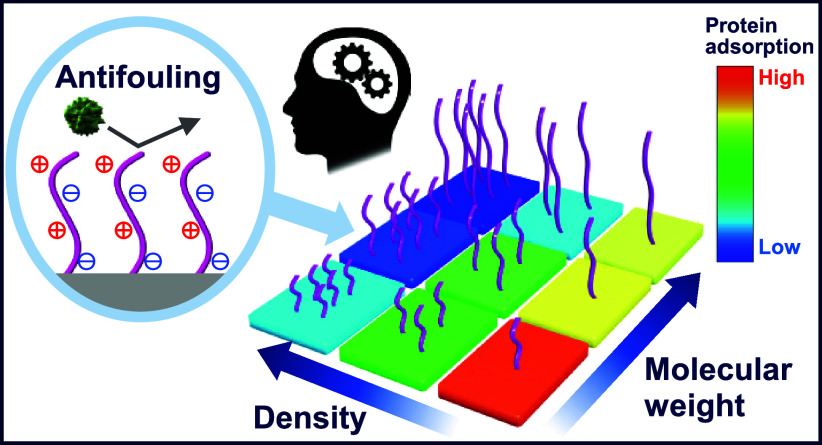

Constructing antifouling surfaces is a crucial technique for optimizing the performance of devices such as water treatment membranes and medical devices in practical environments. These surfaces are achieved by modification with hydrophilic polymers. Notably, zwitterionic (ZI) polymers have attracted considerable interest because of their ability to form a robust hydration layer and inhibit the adsorption of foulants. However, the importance of the molecular weight and density of the ZI polymer on the antifouling property is partially understood, and the surface design still retains an empirical flavor. Herein, we individually assessed the influence of the molecular weight and density of the ZI polymer on protein adsorption through machine learning. The results corroborated that protein adsorption is more strongly influenced by density than by molecular weight. Furthermore, the distribution of predicted protein adsorption against molecular weight and polymer density enabled us to determine conditions that enhanced (or weaken) antifouling. The relevance of this prediction method was also demonstrated by estimating the protein adsorption over a wide range of ionic strengths. Overall, this machine-learning-based approach is expected to contribute as a tool for the optimized functionalization of materials, extending beyond the applications of ZI polymer brushes.

## 1 Introduction

Zwitterionic (ZI) polymers are garnering considerable attention in practical applications owing to their ability to impart high hydrophilicity and antifouling properties at the material interface. They are notably used in coatings on various material surfaces, where the adsorption of contaminants (foulant) can impact the performance of the device, such as membranes for water treatment,^1^ biosensors,^2,3^ medical devices,^4^ and drug delivery systems.^5^ The widely studied ZI polymers contain anions (such as phosphorylcholine, sulphobetaine, and carboxybetaine groups) and cations (primarily the ammonium group) pendent to a methacrylic polymer backbone. These ZI polymers possess unique properties that distinguish them from typical non-ionic polymers. The high antifouling properties of ZI polymers in aqueous environments result from the strong electrostatic interaction of the zwitterions with water molecules: they have 6–11 non-freezing and 4–11 intermediate water molecules per unit, which form a strong and thick hydration layer.^6^ This hydration layer inhibits the proximity of the foulant to the material interfaces and increases the Gibbs energy required for adsorption.^7,8^ As a result, ZI polymer brushes enable better stability and antifouling properties than typical hydrophilic polymer brushes [e.g., polyethylene glycol (PEG)], which form a hydration layer through hydrogen bonds. In addition, ZI polymers specifically interact with ions in solution. At low ionic strength in solution, ZI polymers adopt a collapsed conformation, owing to strong intra/interchain electrostatic dipole–dipole interactions.^9,10^ In contrast, as the salt concentration increases, the ions in solution shield this interaction, causing it to shift to an extended conformation. This effect, known as the “antipolyelectrolyte effect”, exhibits an opposite behavior to that of typical polyelectrolytes.^11,12^

Designing an optimum ZI polymer brush that maximizes the antifouling properties in each aqueous environment is an important but challenging subject. Experimental approaches considered the appropriate conditions, such as ionic groups,^13^ aqueous ionic strength,^12,14−16^ pH,^17^ temperature,^18^ and flow rate,^19^ to exhibit excellent antifouling properties. Further, the effect of brush properties, such as polymer density^20^ and molecular weight,^21,22^ on protein adsorption has also been extensively studied via analytical methods, such as quartz crystal microbalance with dissipation monitoring (QCM-D) and surface plasmon resonance (SPR). However, conducting quantitative studies remains difficult because of the complex contribution of various factors with different physical origins to the adsorption phenomenon on polymer brushes. Therefore, no optimal brush structure is established for each water quality, and it is only empirically understood for the correlation between each factor and the adsorption properties. Machine learning (ML) is a powerful approach for such complex experimental systems. ML is a method that can analyze vast amounts of data in a very short period, enabling the prediction of unknown data and the analysis of the importance of descriptors from the learning of existing data. Indeed, in various fields, such as polymer chemistry,^23^ batteries,^24^ and catalytic chemistry,^25^ the discovery of parameters important for performances^26^ and optimization of the elemental composition of materials^27,28^ have been successfully achieved. However, there are surprisingly few validations using ML for understanding protein adsorption on ZI polymer brush surfaces. To the best of our knowledge, only a few reports have predicted the amount of protein adsorption from brush-related descriptors.^29,30^ Notably, Liu et al. conducted ML using 94 entries to study the correlation between the polymer layer thickness in the dry state and the amount of protein adsorption.^29^ They obtained the following important conclusions: among the descriptors, the polymer layer thickness most considerably contributes to protein adsorption, and an optimal polymer layer thickness exists that minimizes adsorption. Some experimental approaches through contact angle measurement^18^ and serum adsorption test confirmed the existence of an optimum polymer thickness (typically in the range of 30–60 nm)^31^ that demonstrated the applicability of ML to the design of antifouling interfaces.

Meanwhile, the integration of the ML and the design of the ZI polymer brushes are still in their early stages, and several aspects remain unclear. Initially, the external environment outside the polymer layer (i.e., the properties of the protein solution and the operating conditions, such as the flow rate) was largely overlooked. Furthermore, the characteristics of the polymer layer, which are the most important factors influencing the adsorption properties, are not sufficiently considered. Indeed, most previous studies (in experimental approaches^31,32^ and material informatics^29,30^) used the layer thickness in the dried state obtained by ellipsometry as the descriptor of the polymer layer. However, such thickness information does not adequately describe the polymer morphology in the swelling state. For instance, even if the layer thickness in the dry state is comparable, the layer thickness and polymer molecular weight on swelling may considerably differ.^33^ Therefore, to understand the fouling phenomenon and design an optimized antifouling surface, a comprehensive ML-based platform is required that incorporates the detailed features of the polymer layer and external environment.

**Figure 1:**
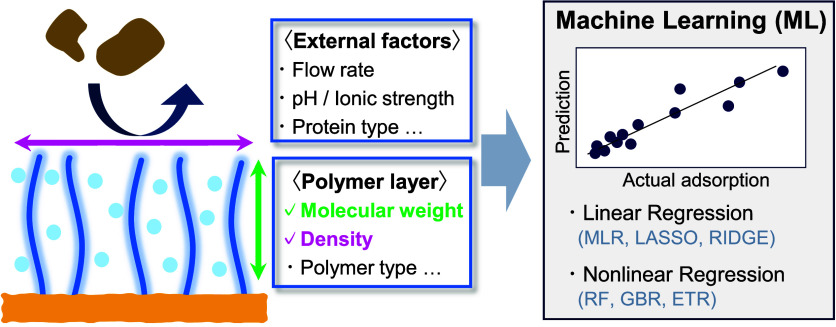
Schematic of this research. In addition to external factors, such as flow velocity and pH/ionic strength, our ML model considers molecular weight and density as the polymer layer descriptors.

Herein, we developed a ML model for the fouling phenomenon of single-component protein adsorption on ZI polymer brushes. This model can evaluate the influence of the molecular weight and density of the brushes. In addition to a detailed examination of the brush structure, the model introduces descriptors for external factors to identify the key factors involved in protein adsorption. Herein, the impact of the molecular weight and density on the ZI polymer brush is separately assessed for the first time. As a result, the amount of protein adsorption (Figure 1) was predicted with high accuracy. On the basis of the constructed model, we identified the brush conditions that enhance antifouling properties.

## 2 Data Set and Methods

### 2.1 Data Set Construction

Data samples were collected from previously reported literature that provided the density of the ZI polymer, molecular weight, and thickness, along with the corresponding protein adsorption. For the literature in which exact values were not mentioned in the text, the values were extracted from the graphs. We further excluded data where protein adsorption on the substrate (without polymer) was not stated and where protein composition in the solution was unclear (e.g., adsorption data using fetal bovine serum). As a result, 12 descriptors were considered, as shown in Table 1. Furthermore, correlation *r*_*ab*_ between descriptors *x* and *y* was assessed using the following Pearson coefficients:^34^
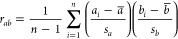
1where *a*_*i*_ and *b*_*i*_ are *i*th sample values, *a̅* and *b̅* are the means of the sample values, and *s*_*a*_ and *s*_*b*_ are the standard deviations of the sample values of descriptors for *a* and *b*, respectively.

**Table 1:** Summary of the Descriptors Used in This Study

descriptor	description
Density	density of the zwitterionic polymer (nm^–2^)
Mn	number-average molecular weight of the zwitterionic polymer
Pol_Type	type of zwitterionic polymer^a^
Sub_Ad	protein adsorption (ng cm^–2^)
Thickness	thickness of the polymer brush in the dry state (nm)
Ionic Strength	ionic strength of the solution (mM)
Temp	temperature of the solution (°C)
pH	pH of the solution
Mpro	molecular weight of the protein (Da)
Charge	charge of the protein^b^
Flow Rate	flow rate of the solution (mL min^–1^)
Pro_Conc	protein concentration (g/L)

a Types of zwitterionic polymers were defined as the amount of hydrated water per monomer unit (Figure S1 of the Supporting Information).

b The charge of each protein was defined as pI – pH using the isoelectric point (pI) of the protein.

### 2.2 ML Models and Interpretations

All ML processes were performed using Scikit-Learn Library 1.3.0 in Python 3.11.5. The Shapley additive explanations (SHAP) value for each feature was estimated using SHAP Library 0.42.1. Herein, we examined the performance of six algorithms: multiple linear regression (MLR), least absolute shrinkage and selection operator regression (LASSO), ridge regression (Ridge), random forest regression (RFR), gradient-boosted regression (GBR), and extra-tree regression (ETR). The hyperparameters for each algorithm were first optimized using the randomized search cross-validation (GridSearchCV) technique from the range of values provided in Table S1 of the Supporting Information to prevent underfitting and overfitting of the model. The 123 data samples were randomly divided into a training set (103 data samples) and a test set (20 data samples). The training set was used to train each model, and the test set was used to validate the accuracy of the trained model. To quantitatively evaluate the predictive accuracy of each regression model, the root-mean-square error (RMSE) and coefficient of determination score (*R*^2^) were compared. The RMSE and *R*^2^ are obtained by the following equations:
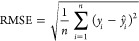
2
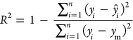
3where *n* is the total number of data, *ŷ*_*i*_ is the predicted value of the *i*th sample, *y*_*i*_ is the measured protein adsorption amount, and *y*_m_ is the mean value of all corresponding true values in the training set. A small value of RMSE indicated a better prediction by the model, and a value of *R*^2^ close to 1.0 implied a better match between the measured and predicted data.

To enhance model interpretability, we further implemented SHAP. The SHAP is a method developed by Lundberg and Lee^35^ based on coalition game theory to describe the output of ML models. Using SHAP values, we can quantify the contribution of each descriptor to the predicted value from the constructed model. The SHAP value for an input feature *x* (out of a total of *n* input features), given a prediction *p* from the constructed ML model, is represented by the following equation:
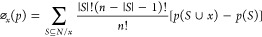
4where *S* represents the subset for each feature without feature *x*, *p*(*S* ∪ *x*) represents the predictions through ML considering feature *x*, and *p*(*S*) represents the predictions without considering feature *x*. Herein, SHAP analysis was conducted to analyze the impact of each descriptor on protein adsorption. The relative importance of each descriptor was quantitatively compared on the basis of the absolute SHAP values.

## 3 Results and Discussion

### 3.1 Data Set of Protein Adsorption for the ZI Polymer Brush

Whitesides et al. previously collected correlations between non-ionic molecular structures and fouling properties,^36^ and ML-based analysis using those data samples was also reported.^37−39^ However, the data set for ZI polymers remains unconstructed. Therefore, this study began by constructing a series of data samples that summarized protein adsorption with brush properties (i.e., density, molecular weight, and polymer type), solution properties (i.e., pH, temperature, concentration, protein characteristics, and ionic strength), and operating conditions (i.e., flow rate). To construct a reliable data set from the literature, we need to consider the homogeneity of the polymer brushes. When brushes are formed by typical radical polymerization, the distribution of the molecular weight is wide; thus, we cannot properly assess the effects of the molecular weight and density. Therefore, all cited literature controls the molecular weight by introducing precisely controlled polymerization, such as atom transfer radical polymerization (ATRP). In the controlled grafting to method, a brush can be formed by directly bonding the length-controlled polymer to the surface through terminal functional groups (e.g., thiol groups). In the controlled grafting from method, surface-initiated ATRP (SI-ATRP) is primarily applied to the substrate.The SI-ATRP allows for the indirect identification of the polymer molecular weight using sacrificial initiators,^40−42^ and the polymer density is determined from the amount of introduced initiator.

On the basis of these experimental reports, we ultimately obtained 125 data samples without missing values, as provided in Table S2 of the Supporting Information. We removed entries 1 and 2 from the data set because protein adsorption on the substrate was extremely large and difficult to properly assess (see the Supporting Information and Figure S2 of the Supporting Information). Consequently, 123 data samples were used for ML.

**Figure 2:**
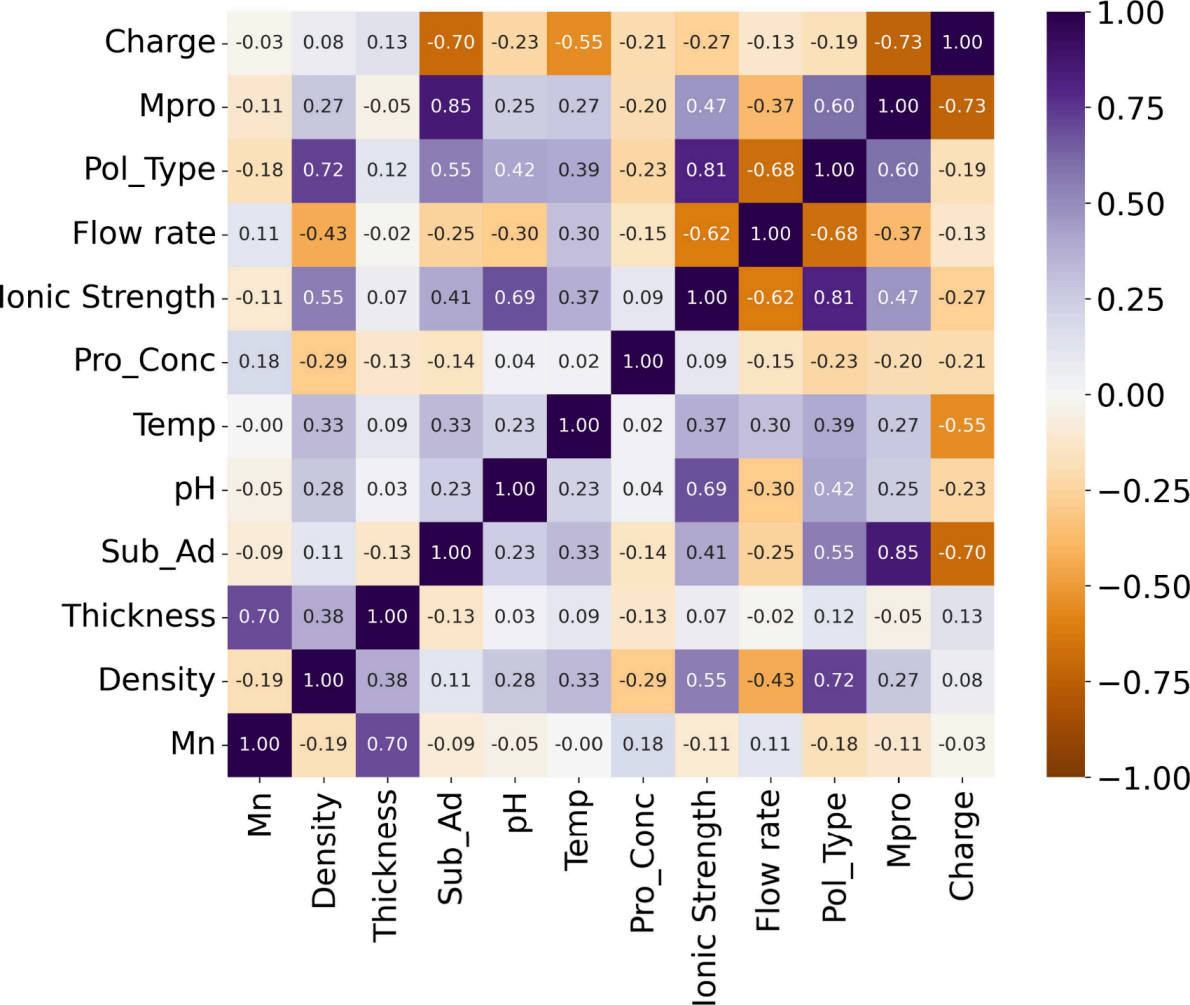
Heatmap of the Pearson coefficients among 12 selected features. The Pearson correlation coefficient ranges from −1 to 1, where 1 represents an absolute positive correlation and −1 represents an absolute negative correlation. Each feature is described in Table 1.

As shown in Table 1, the data set contains five descriptors for the polymer brush properties and seven descriptors for the solution/operating conditions. Before implementing the ML model, we calculated the Pearson correlation coefficients for each feature. A strong correlation between the two features can increase the difficulty of the training model and may affect prediction accuracy. Thus, when the correlation coefficient between two features exceeds 0.9^43^ or 0.95,^30,44^ the feature that has a high correlation with the prediction target is typically retained and the other feature is deleted. Figure 2 shows that none of the 12 features used in this study has a strong correlation beyond this threshold; thus, we did not delete any descriptors from this study.

### 3.2 Comparison and Selection of the Regression Methods

**Figure 3:**
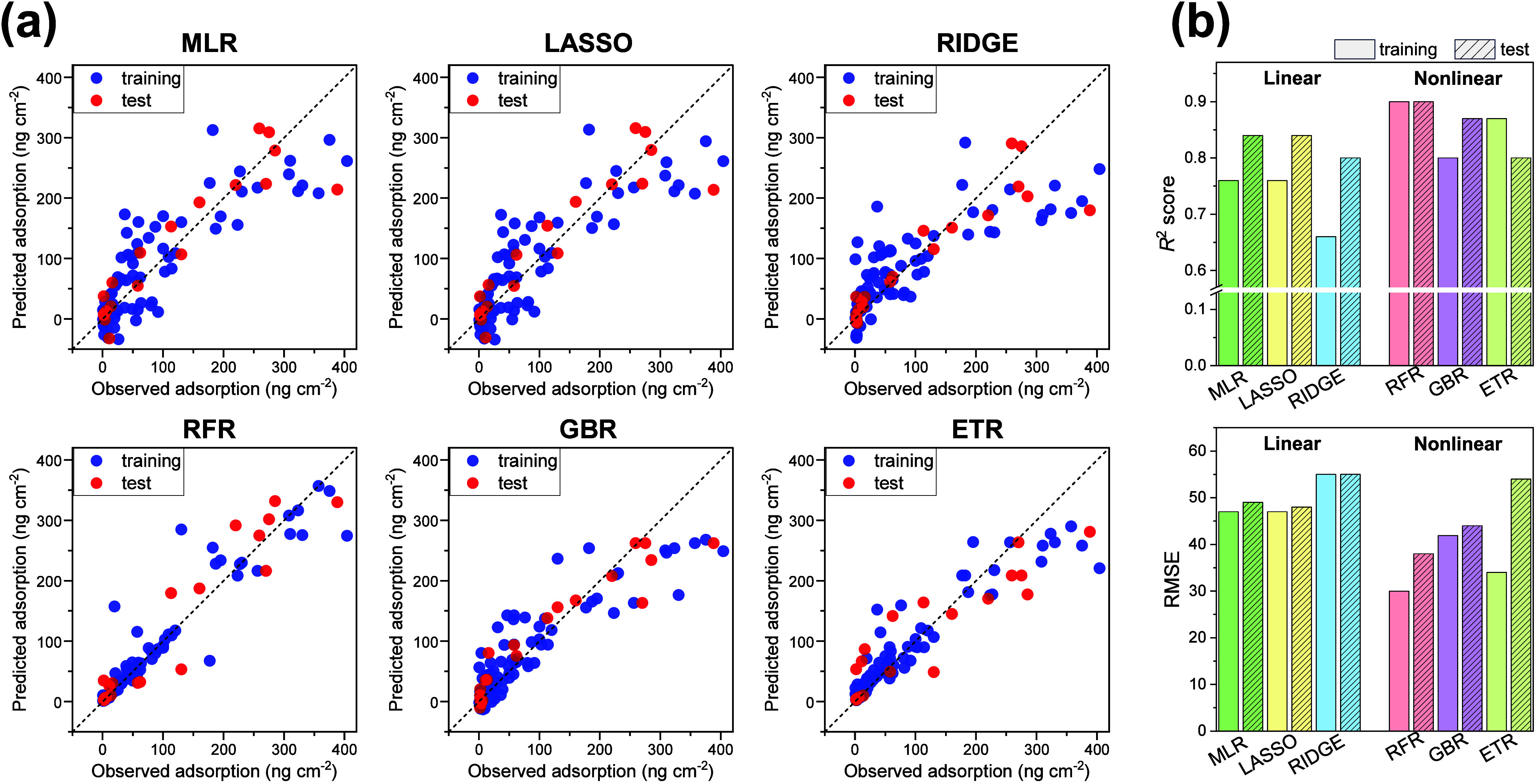
(a) Images of the prediction performance of ML using three types of linear regressions and three types of nonlinear regressions. (b) *R*^2^ and RMSE values of each algorithm in predicting the amount of adsorbed protein. Values for test data are shown with diagonal lines.

Three linear regression algorithms (MLR, LASSO, and RIDGE) and three decision tree-based nonlinear regression algorithms (RFR, GBR, and ETR) were performed using the previously constructed training and test data sets. Initially, ML was performed using all of the descriptors presented in Table S2 of the Supporting Information. Note that the thickness, molecular weight, and density are all included in the first run. However, thickness is the dependent variable of molecular weight and density: *M*_n_ = *h*ρ*N*_A_/σ, where *M*_n_ is the molecular weight, *h* is the dry brush thickness, ρ is the dry polymer density, *N*_A_ is Avogadro’s number, and σ is the chain density.^41,42,45^ Elimination of this overlap is considered in sections 3.4 and 3.5. Figure 3a presents the results of the analysis obtained from each algorithm, showing that the nonlinear regression algorithms have high accuracy. This suggests that the adsorption phenomenon cannot be represented by a simple linear sum of each descriptor. Figure 3b confirmed the superiority of the tree-based regression algorithms, presenting the efficiency of each algorithm as *R*^2^ scores and RMSE. In a previous report that applied ML, similar trends are presented to protein adsorption on non-ionic polymer brushes.^30^Figure 3b also indicates that RFR is the most suitable algorithm in this study; thus, RFR was used for further validation.

### 3.3 SHAP-Analysis-Based Importance Estimation of the Descriptors

**Figure 4:**
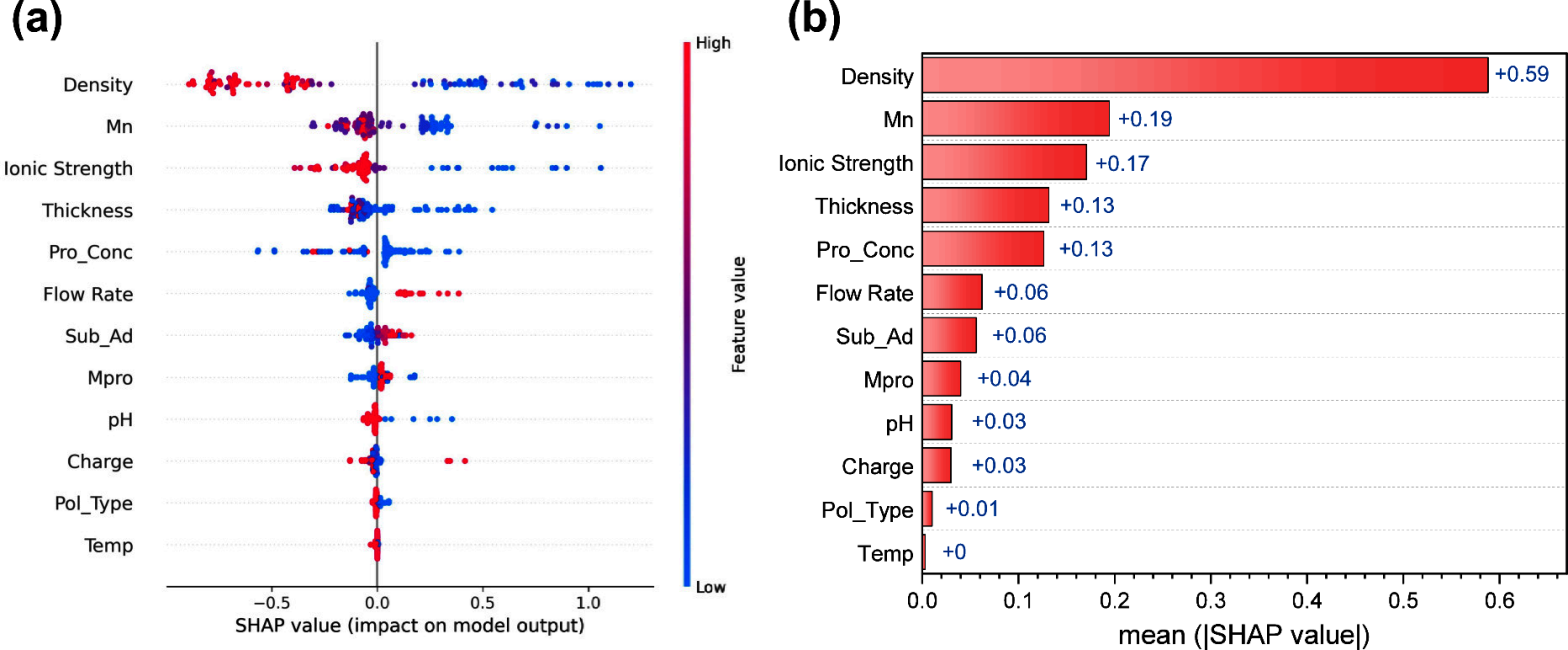
(a) SHAP plot for protein adsorption in the RFR model. Colors from red to blue represent the feature values from more to less. (b) Estimated importance (mean SHAP value) of considered descriptors.

Next, we quantitatively evaluated the importance of each descriptor in the built RFR model using SHAP. Large SHAP values are strongly weighted in the prediction; therefore, the magnitude of positive and negative SHAP values indicates the importance of the feature. Figure 4a shows that the descriptors of polymer density, molecular weight, and ionic strength negatively contributed to protein adsorption (i.e., higher values led to lower adsorption). In contrast, the flow rate and substrate adsorption positively contributed. These results align with previous experimental findings. For instance, Zhang et al. explicitly demonstrated an inverse correlation between the ionic strength and protein adsorption under various ionic species.^12^ Similarly, Amoako et al. showed that protein adsorption increased when the ZI polymer was under shear stress at high flow rates.^19^ The consistency of the SHAP analysis with these experimental facts supports the validity of the ML model constructed in this study.

Furthermore, we calculated the important scores for each descriptor from the absolute average of the SHAP values (Figure 4b). Upon examination of the descriptors with high importance, polymer density had the highest score (+0.59), almost 3 times higher than the molecular weight *M*_n_ (+0.19). Both parameters effectively inhibit protein adsorption; however, the antifouling mechanisms are presumed to be different. High polymer density promotes the formation of a robust hydration layer. As a result, high density increases osmotic pressure for protein insertion into the polymer layer. On the other hand, high polymer molecular weight prevents the protein adsorption by increasing the distance between the substrate surface and solution. Therefore, our results indicate that the formation of rigid hydration and the increase in osmotic pressure owing to high brush density are more important than the increase in diffusion distance by high molecular weight. In addition, solution/protein properties (Temp, Charge, pH, and Mpro) and the type of ZI polymer (Pol_Type) were found to have a small influence (<0.05) on protein adsorption. The minimal influence of the solution temperature and pH may be because the referred literature adopted the applicable pH and temperature ranges^42^ of the ZI polymers. Meanwhile, the small influence of the monomer structure (Pol_Type) and protein properties (Charge and Mpro) differs from the results acquired by ML for non-ionic hydrophilic polymers,^29,30^ indicating that this is a property specific to the ZI polymer brushes. This is likely because typical hydrophilic polymers, such as PEG, use their own steric repulsion,^8^ whereas the ZI polymers operate by the hydration layer on the polymer brush. Ultimately, we excluded these parameters and did not consider them in the later ML modeling. Moreover, Figure 4b also shows that the descriptors, such as ionic strength (+0.17), protein concentration (+0.13), and flow velocity (+0.06), have an intermediate importance. Thus, for the first time, the importance of the interface, operating conditions, and external environment for protein adsorption was quantified, which has not been qualitatively understood thus far.

### 3.4 Consideration of the Descriptor for the Polymer Brush

**Figure 5:**
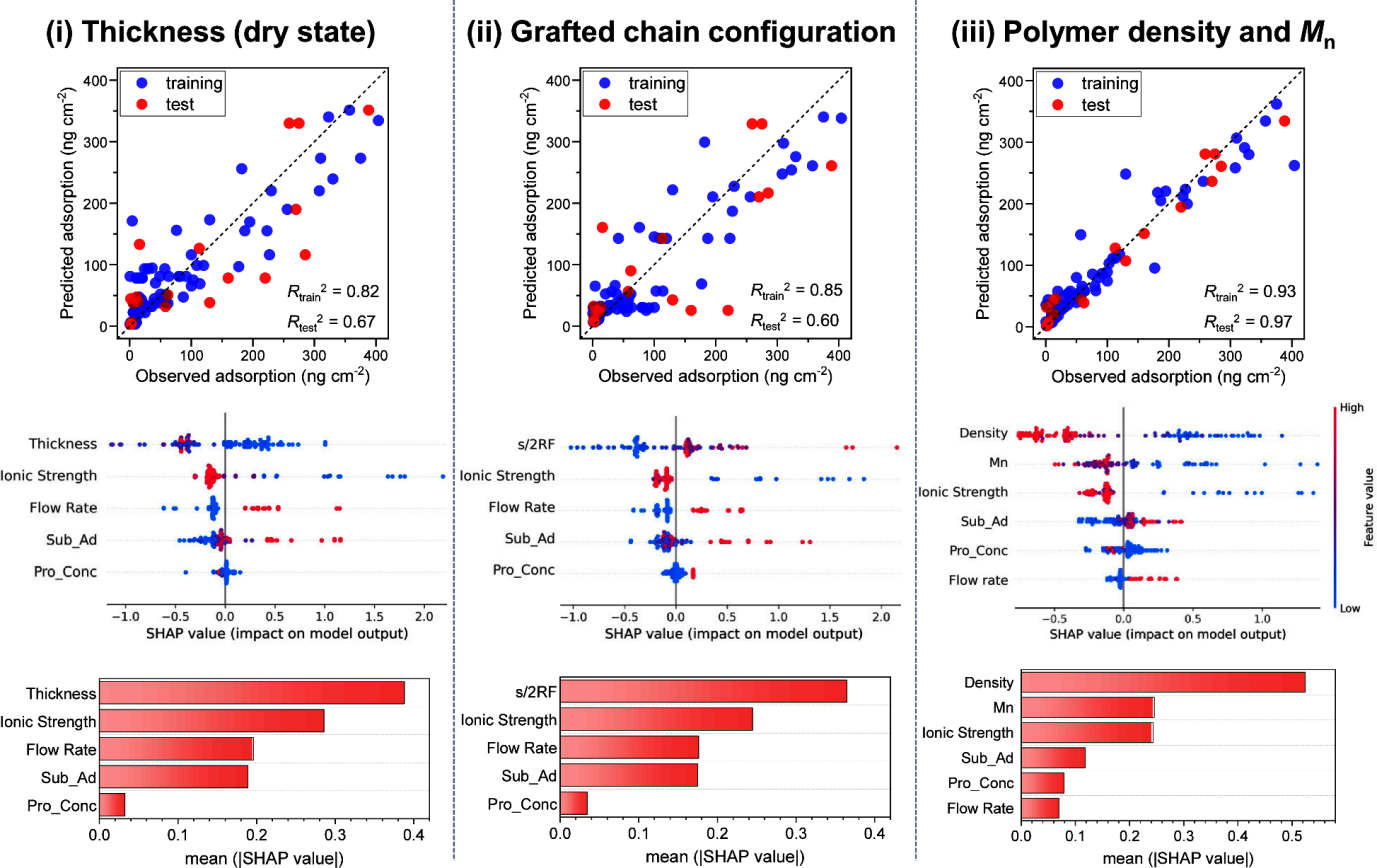
Results of ML by the RFR model using (i) layer thickness in the dry state, (ii) s/2*R*_F_ value, and (iii) polymer density and molecular weight as the descriptors for the grafted ZI polymer. The quantity of adsorption without polymer, protein concentration, ionic strength, and flow rate were used as external descriptors.

In addition to the molecular weight and density, the Flory radius is also used as a length parameter of the brush polymer.^46−48^ The grafted chain configuration, which is estimated from the Flory radius, is a well-known indicator of the degree of the brush state
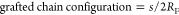
5where *R*_F_ is the Flory radius of the hydrated polymer, expressed as *R*_F_ = *lN*^3/5^ (*l* is the monomer length of ≈0.3 nm and *N* is the degree of polymerization),^49^ and *s* is the average distance between grafted polymers, which is expressed as the inverse of the square root of the polymer density. Generally, the polymer chain forms a mushroom structure at *s*/2*R*_F_ ≫ 1 and a brush structure at *s*/2*R*_F_ ≪ 1, and a weakly overlapped structure is formed between mushrooms and brushes.^50^ We further investigated the most appropriate descriptor for the polymer brush structure using the built RFR model. In the RFR model, (i) conventionally investigated polymer thickness (drying state), (ii) s/2*R*_F_ value, and (iii) density and *M*_n_ were considered as the descriptors for the polymer brush. In addition, Sub_Ad, Pro_Conc, Ionic Strength, and Flow Rate were used as external descriptors based on the importance scores shown in Figure 4b. As shown in Figure 5, case i had a low prediction accuracy [*R*^2^ for training data (*R*_train_^2^) = 0.80 and *R*^2^ for test data (*R*_test_^2^) = 0.63]. This confirms our assumption regarding the uncertainty of the thickness parameter, as already mentioned in Figure 1a. Considerable improvement in prediction performance was not achieved even when the *s*/2*R*_F_ value was used as a brush descriptor (*R*_train_^2^ = 0.85 and *R*_test_^2^ = 0.60). This is because the descriptor *s*/2*R*_F_ cannot identify specific molecular weights and densities. In comparison to cases i and ii, the best prediction accuracy (*R*_train_^2^ = 0.93 and *R*_test_^2^ = 0.97) was achieved in case iii, which used polymer density and molecular weight as the descriptors for the polymer brush. Here, there are still some outliers in the training data that are not due to the polymer configuration but due to differences in the environment of each experiment (see the Supporting Information and Figure S3 of the Supporting Information). In case iii, the trend of the SHAP plots and the estimated importance distribution is similar to that when all descriptors are considered in Figure 4; the importance of density is the highest (2.1 times higher than that of the molecular weight). These results show that thickness or grafted chain configuration is insufficient to accurately represent the properties of polymer brushes; thus, polymer density and molecular weight are required to estimate protein adsorption.

### 3.5 Dependence of Protein Adsorption upon the Brush Density and Molecular Weight

**Figure 6:**
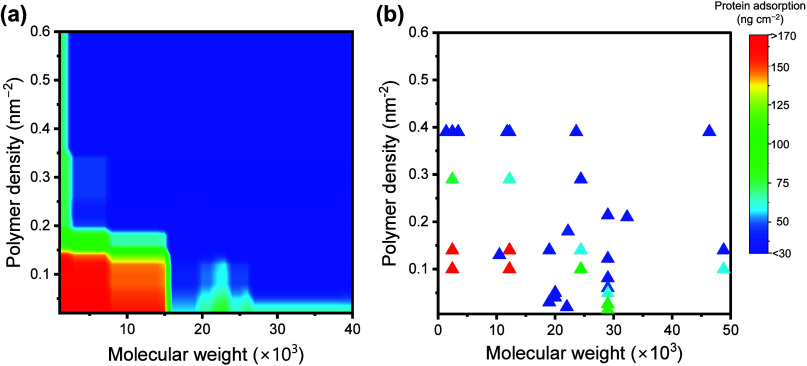
(a) Visualization for mapping of protein adsorption against the molecular weight and density of the ZI polymer brushes. The degree of protein adsorption was predicted using a trained RFR model, and the descriptors Ionic Strength, Sub_Ad, Pro_Conc, and Flow Rate were fixed. For each mapping, the molecular weights up to 40 000 g mol^–1^ and densities up to 0.6 chains nm^–2^ were investigated, providing 1200 predicted data. (b) Color map of experimental data samples within a high ionic strength range of 100–200 mM. The data were extracted from the constructed data set.

The correlation between surface properties and adsorption was investigated using a trained RFR model to clarify the effect of the molecular weight and density of the brush polymer on protein adsorption. Herein, the amount of adsorbed protein was predicted for 1200 conditions at various polymer molecular weights (*M*_n_ ∼ 40 000, with the interval = 1000) and densities (σ ∼ 0.6, with the interval = 0.02). First, we predicted protein adsorption by fixing the parameters at Ionic Strength = 150 mM, Sub_Ad = 450 ng cm^–2^, Pro_Conc = 1 g L^–1^, and Flow Rate = 0.01 mL min^–1^. Figure 6a shows the mapping of the predicted amounts of protein adsorption against the molecular weights and densities of the polymer brushes. The predicted adsorption distribution cannot be interpreted simply in terms of differences in polymer configurations, as previously described. In fact, the boundary of *s*/2*R*_F_ = 1 differs from the adsorption trend (Figure S4 of the Supporting Information). Therefore, adsorption on ZI polymer brushes is a complex phenomenon that involves the influence of external hydration layers beyond the brush structures.

In the prediction mapping, blocky color changes were generated, probably as a result of the limited number of data samples. However, mapping well expressed the features of experimental facts, and prediction can be used for further investigation. Figure 6b shows the color map of the grouped data samples with a high ionic strength range of 100–200 mM. Unlike the prediction, the parameter values (e.g., flow rate and protein concentration) differ for each experimental data. Nevertheless, the experimental data exhibited a robust correlation with the predicted outcomes, validating the predictions. Further, Figure 6b shows the limitations of experimentally feasible surfaces. Notably, no data samples were observed for surface densities with σ > 0.4 chains nm^–2^, indicating that such surface densities are difficult to achieve experimentally as a result of steric hindrance by graft polymers.

**Figure 7:**
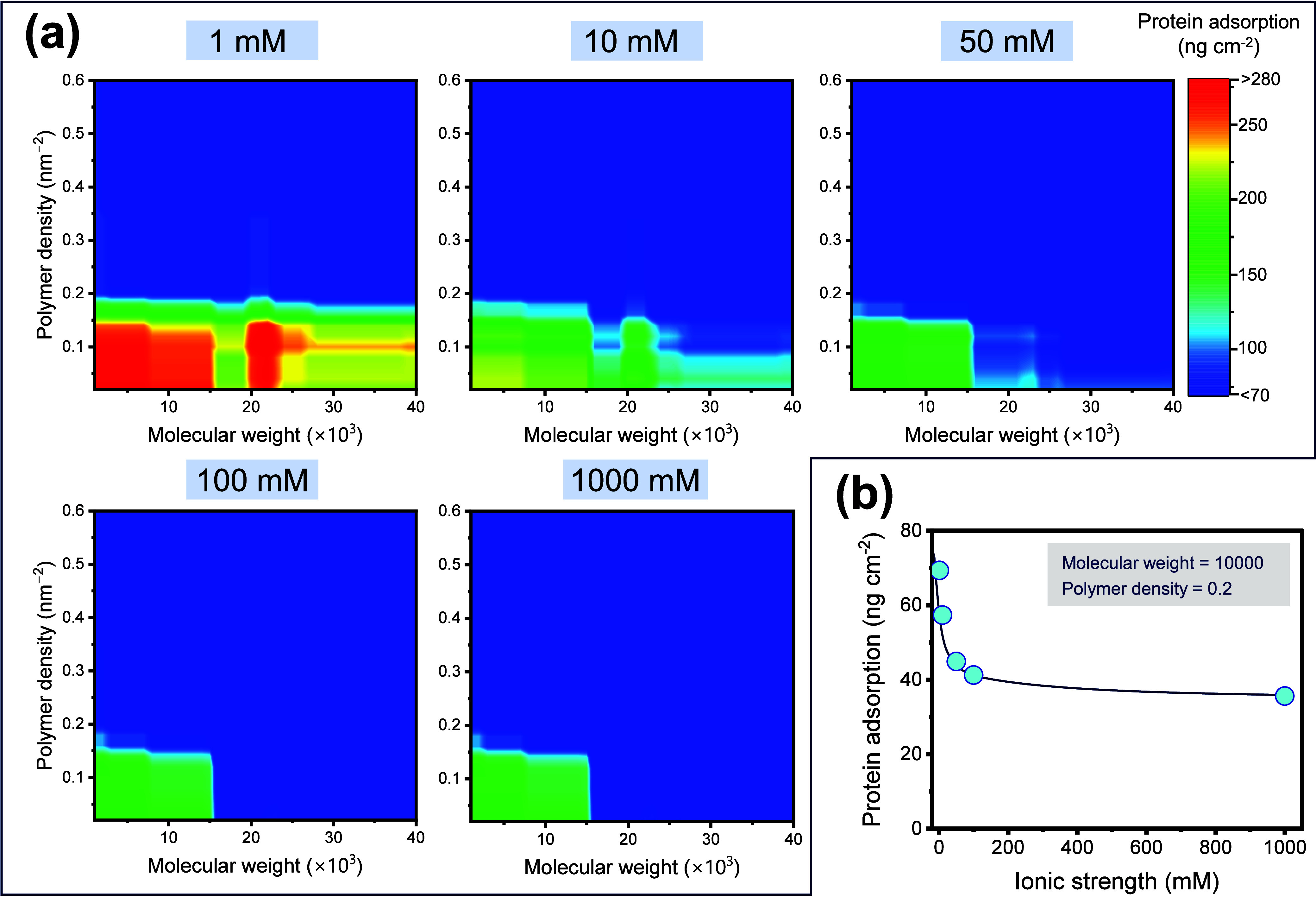
(a) Ionic strength dependence of the mapping protein adsorption. The descriptors are fixed as follows: Sub_Ad = 450 ng cm^–2^, Pro_Conc = 1 g L^–1^, and Flow Rate = 0.01 mL min^–1^. (b) Predicted protein adsorption on the surface of ZI polymer brushes as a function of the ionic strength. The molecular weight is fixed at 10 000, and the polymer density is fixed at 0.2.

ML-aided prediction of protein adsorption can help to understand adsorption properties beyond current experimental facts or extract desirable surface conditions in practical environments. Thus, we further predicted protein adsorption on ZI polymer brushes concerning the molecular weight and density under various water environments. Here, the influence of the ionic strength, which shows high importance in case iii of Figure 5, was investigated. The consideration of the ionic strength is particularly crucial in the application of antifouling porous membranes for water treatment. Generally, groundwater and surface water have millimolar ranges of ionic strength, whereas seawater has approximately 700 mM. Therefore, we examined ionic strengths in the range of 1–1000 mM (Figure 7a).

Overall, the amount of adsorption tended to be larger at a lower ionic strength, which reproduces the decrease in the antifouling properties associated with the previously mentioned antipolyelectrolyte effect. With focus on the dependence of protein adsorption upon the molecular weight and density, the degree of influence of graft density is clearly greater than the influence of the molecular weight. Notably, favorable antifouling performance is exhibited in the region of densities above 0.2. We also observed the regions of high adsorption at low graft densities (<0.15) and moderate *M*_n_ (20 000–23 000). This region with high protein adsorption may correspond to the “hot spot” that have been newly discovered by an experimental approach.^51^ Under such conditions, a decrease in ionic strength causes the hydration structure to collapse, and the distance between the polymer chains exceeds the protein size. Consequently, the protein is inserted within the three-dimensional space of the ZI polymer layer, and adsorption occurs via electrostatic interactions. However, all adsorptions at *M*_n_ > 15 000 can efficiently decrease with the formation of a hydration layer as a result of an increase in ionic strength. Meanwhile, protein adsorption is significant in areas with low densities and molecular weights (σ < 0.15 and *M*_n_ < 15 000). The adsorption is not sufficiently decreased even in a high ionic strength environment, attributable to the existence of a defective surface with an exposed substrate. In such a surface, the protein can directly adsorb onto the substrate and the antifouling effect through the hydration layer is not effectively exhibited.

With focus on the correlation between protein adsorption and ionic strength, it is suggested that ZI polymer brushes effectively exhibit antifouling properties at an ionic strength over 100 mM. This is also evident from Figure 7b, which shows the ionic strength dependence of protein adsorption under specific molecular weights and densities. This result agrees with previous reports that experimentally show that ZI brushes can effectively resist protein adsorption in the range of ionic strength above 100 mM.

Overall, the findings gathered from the application of ML to ZI polymer brushes are as follows: (1) The RFR was chosen as the best model to maximize the prediction performance. The positive or negative contribution of each descriptor to protein adsorption agreed with the experimental facts that supports the reliability of the built model. (2) The effects of the brush density and molecular weight on protein adsorption were separately evaluated for the first time. The results present that protein adsorption is more strongly influenced by the density than by the molecular weight. (3) Several descriptors (thickness, *s*/2*R*_F_ value, and density with *M*_n_) for the brush structure were investigated. The highest prediction performance was achieved using density and *M*_n_. (4) Visualization on the mapping of protein adsorption against brush density and molecular weight was provided for brush conditions that increase or decrease antifouling properties. Further, the effect of the ionic strength on antifouling properties was investigated to demonstrate the relevance of this study.

As demonstrated in this study, ML is useful for understanding the overall adsorption phenomena from limited experimental data and determining the optimal brush structure in each environment. However, this research is still in its early stages and currently has several limitations. For example, more data samples are required to predict specific water environments. An increase in the number of data samples would allow for detailed analysis of the effects of each factor (such as the protein concentration, protein species, and flow rate) on adsorption behavior. In addition, the antifouling performance of ZI polymers varies with anion/cation moieties in the polymers or ion species in the solution, but their contributions were not considered in this study. Further advanced predictions incorporating these factors will be realized in the future, with the expansion of relevant experimental data.

## 4 Conclusion

Herein, we initially gathered a data set on protein adsorption on the ZI polymer brushes from the literature sources. This data set includes details about brush structures (molecular weight, density, thickness, polymer type, and substrate characteristics), solution conditions (pH, ionic strength, temperature, and protein characteristics), and control conditions (flow rate) that correspond to protein adsorption. Three linear (MLR, LASSO, and RIDGE) and decision-tree-based nonlinear (RFR, GBR, and ETR) regressions were applied to compare prediction performance. The RFR was chosen as the primary ML model because it showed the highest *R*^2^ value (*R*^2^ = 0.9 for training and test data) and the lowest RMSE value. The SHAP analysis for the constructed RFR model showed that polymer density, molecular weight, ionic strength, and polymer layer thickness negatively contributed. The flow rate and adsorption on the substrate positively contributed to the overall protein adsorption. This agrees well with previous experimental reports and supports the validity of the model. Polymer density is shown to have the highest importance, which is 2–3 times higher than that of the molecular weight by comparing the importance of each descriptor via SHAP analysis. In addition, the thickness, grafted chain configuration, and density with *M*_n_ were compared as the descriptors of the polymer brush, and the density with *M*_n_ provided the best prediction in the RFR model. Furthermore, the trained ML model was used to produce a prediction mapping for protein adsorption against the molecular weight and graft density. This mapping allowed for the determination of regions with enhanced antifouling properties. Finally, the effect of the ionic strength on antifouling properties was investigated to demonstrate the relevance of this study.

To the best of our knowledge, this is the first report to accurately estimate the contribution of density and molecular weight to protein adsorption using a ML-based approach. This work quantitatively evaluated the importance of the polymer brush to the antifouling properties, which was empirically not understood until now. Although this study focuses only on the ZI polymers based on a limited number of data sets, the approach may be applicable investigating various brush interfaces and could help for designing future antifouling surfaces.

## Supporting Information Available

List of hyperparameters for ML algorithms, full data set for ML that encompasses brush and external conditions and consequential protein adsorption, and results of ML using all data samples without trimming (PDF)
