# Supplementary material for: Machine-Learning-Aided Understanding of Protein Adsorption on Zwitterionic Polymer Brushes
Source: ACS Appl Mater Interfaces. 2024 May 3;16(19):25236–45. doi: 10.1021/acsami.4c01401 (PMC11103666; doi:10.1021/acsami.4c01401)
Supplement: Supplementary file 1 — am4c01401_si_001.pdf [file am4c01401_si_001.pdf]

Supporting Information

Machine Learning-aided Understanding of Protein Adsorption on Zwitterionic Polymer Brushes

Hiroto Okuyama*, Yuuki Sugawara, Takeo Yamaguchi*

Laboratory for Chemistry and Life Science, Tokyo Institute of Technology, Yokohama 226-8501, Japan.

*Corresponding Author: okuyama.h.ae@m.titech.ac.jp
yamag@res.titech.ac.jp

Table S1. List of hyperparameters for machine learning algorithms.

Category	Algorithm	Hyperparameters
Linear regression	MLR	–
	LASSO	$10^{-6} \leq \alpha \leq 10^{-3}$
	Ridge	$10^{-6} \leq \alpha \leq 10^{-2}$
Nonlinear regression	RFR	n_estimators ∈ [3, 5, 10], max_features ∈ [5, 7, 9, 12], min_samples_split ∈ [2, 5, 10], max_depth ∈ [5, 10, 50], min_samples_leaf ∈ [1, 2, 4]
	GBR	n_estimators ∈ [3, 5, 10], max_features ∈ [5, 7, 9, 12], learning_rate ∈ [0.01, 0.1, 0.5], min_samples_split ∈ [2, 5, 10], max_depth ∈ [5, 10, 50], min_samples_leaf ∈ [1, 2, 4], subsample ∈ [0.2, 0.5, 1.0], min_weight_fraction_leaf ∈ [0.1, 0.2, 0.5]
	ETR	n_estimators ∈ [3, 5, 10], max_features ∈ [5, 10, 20, 30], min_samples_split ∈ [2, 5, 10], max_depth ∈ [5, 10, 50], min_samples_leaf ∈ [1, 2, 4], min_weight_fraction_leaf ∈ [0.01, 0.1, 0.3, 0.5]

Table S2. Dataset of 125 experimental data for machine learning.

Entry	Protein Adsorption (ng/cm^2)	Mn	Density	Thickness	Sub_Ad	pH	Temp	Pro_Conc	Ionic Strength	Flow rate	Pol_Type	Mpro	Charge	Ref.
1	1800	12000	0.74	11.04	3350	7.4	37	1	150	0	5.8	66000	−2.4	1
2	3100	12000	0.14	2.15	3350	7.4	37	1	150	0	5.8	66000	−2.4	1
3	220	12200	0.1	1.5	410	7.4	23	0.05	150	0	9.4	340000	−1.6	2
4	160	12200	0.14	2	410	7.4	23	0.05	150	0	9.4	340000	−1.6	2
5	50	12200	0.29	4.4	410	7.4	23	0.05	150	0	9.4	340000	−1.6	2
6	10	12200	0.39	5.9	410	7.4	23	0.05	150	0	9.4	340000	−1.6	2
7	55	48800	0.1	2.5	410	7.4	23	0.05	150	0	9.4	340000	−1.6	2
8	11	48800	0.14	2.9	410	7.4	23	0.05	150	0	9.4	340000	−1.6	2
9	87	24400	0.1	2.3	410	7.4	23	0.05	150	0	9.4	340000	−1.6	2
10	40	24400	0.14	4.4	410	7.4	23	0.05	150	0	9.4	340000	−1.6	2
11	285	2440	0.1	1	410	7.4	23	0.05	150	0	9.4	340000	−1.6	2
12	227	2440	0.14	1	410	7.4	23	0.05	150	0	9.4	340000	−1.6	2
13	100	2440	0.29	1.15	410	7.4	23	0.05	150	0	9.4	340000	−1.6	2
14	15	2440	0.39	2.2	410	7.4	23	0.05	150	0	9.4	340000	−1.6	2
15	308	12200	0.1	1.5	570	7.4	23	0.1	150	0	9.4	340000	−1.6	2
16	230	12200	0.14	2	570	7.4	23	0.1	150	0	9.4	340000	−1.6	2
17	62	12200	0.29	4.4	570	7.4	23	0.1	150	0	9.4	340000	−1.6	2
18	13	12200	0.39	5.9	570	7.4	23	0.1	150	0	9.4	340000	−1.6	2

19	58	48800	0.1	2.5	570	7.4	23	0.1	150	0	9.4	340000	−1.6	2
20	19	48800	0.14	2.9	570	7.4	23	0.1	150	0	9.4	340000	−1.6	2
21	100	24400	0.1	2.3	570	7.4	23	0.1	150	0	9.4	340000	−1.6	2
22	59	24400	0.14	4.4	570	7.4	23	0.1	150	0	9.4	340000	−1.6	2
23	25	24400	0.29	7.65	570	7.4	23	0.1	150	0	9.4	340000	−1.6	2
24	375	2440	0.1	1	570	7.4	23	0.1	150	0	9.4	340000	−1.6	2
25	310	2440	0.14	1	570	7.4	23	0.1	150	0	9.4	340000	−1.6	2
26	76	2440	0.29	1.15	570	7.4	23	0.1	150	0	9.4	340000	−1.6	2
27	16	2440	0.39	2.2	570	7.4	23	0.1	150	0	9.4	340000	−1.6	2
28	0.675	19000	0.03	0.83	440	7.4	25	1	150	0.05	9.4	66000	−2.4	3
29	4	19000	0.03	0.83	1246	7.4	25	1	150	0.05	9.4	340000	−1.6	3
30	19.5	10472	0.13	1.74	500	7.4	25	1	150	0.05	9.4	66000	−2.4	4
31	49	1380	0.39	0.83	261	7.4	23	0.005	150	0	9.4	340000	−1.6	5
32	10.5	3430	0.39	1.7	261	7.4	23	0.005	150	0	9.4	340000	−1.6	5
33	2.7	11800	0.39	5.9	261	7.4	23	0.005	150	0	9.4	340000	−1.6	5
34	3.5	23600	0.39	11.8	261	7.4	23	0.005	150	0	9.4	340000	−1.6	5
35	1.5	46360	0.39	23.1	261	7.4	23	0.005	150	0	9.4	340000	−1.6	5
36	81.5	1380	0.39	0.83	364	7.4	23	0.05	150	0	9.4	340000	−1.6	5
37	16.5	3430	0.39	1.7	364	7.4	23	0.05	150	0	9.4	340000	−1.6	5
38	10.9	11800	0.39	5.9	364	7.4	23	0.05	150	0	9.4	340000	−1.6	5
39	7.7	23600	0.39	11.8	364	7.4	23	0.05	150	0	9.4	340000	−1.6	5
40	1.95	46360	0.39	23.1	364	7.4	23	0.05	150	0	9.4	340000	−1.6	5

41	63.3	1380	0.39	0.83	404	7.4	23	0.25	150	0	9.4	340000	−1.6	5
42	14	3430	0.39	1.7	404	7.4	23	0.25	150	0	9.4	340000	−1.6	5
43	7.8	11800	0.39	5.9	404	7.4	23	0.25	150	0	9.4	340000	−1.6	5
44	6.2	23600	0.39	11.8	404	7.4	23	0.25	150	0	9.4	340000	−1.6	5
45	3.5	46360	0.39	23.1	404	7.4	23	0.25	150	0	9.4	340000	−1.6	5
46	80	1380	0.39	0.83	435	7.4	23	0.5	150	0	9.4	340000	−1.6	5
47	15.5	3430	0.39	1.7	435	7.4	23	0.5	150	0	9.4	340000	−1.6	5
48	6.6	11800	0.39	5.9	435	7.4	23	0.5	150	0	9.4	340000	−1.6	5
49	6.6	23600	0.39	11.8	435	7.4	23	0.5	150	0	9.4	340000	−1.6	5
50	5.4	46360	0.39	23.1	435	7.4	23	0.5	150	0	9.4	340000	−1.6	5
51	91.7	1380	0.39	0.83	473	7.4	23	1	150	0	9.4	340000	−1.6	5
52	20.6	3430	0.39	1.7	473	7.4	23	1	150	0	9.4	340000	−1.6	5
53	10	11800	0.39	5.9	473	7.4	23	1	150	0	9.4	340000	−1.6	5
54	9.3	23600	0.39	11.8	473	7.4	23	1	150	0	9.4	340000	−1.6	5
55	6.6	46360	0.39	23.1	473	7.4	23	1	150	0	9.4	340000	−1.6	5
56	12.5	1380	0.39	0.83	22.8	7.4	23	0.005	150	0	9.4	14307	3.6	5
57	5.7	3430	0.39	1.7	22.8	7.4	23	0.005	150	0	9.4	14307	3.6	5
58	1.4	11800	0.39	5.9	22.8	7.4	23	0.005	150	0	9.4	14307	3.6	5
59	2.1	23600	0.39	11.8	22.8	7.4	23	0.005	150	0	9.4	14307	3.6	5
60	2.5	46360	0.39	23.1	22.8	7.4	23	0.005	150	0	9.4	14307	3.6	5
61	9.4	1380	0.39	0.83	55.6	7.4	23	0.05	150	0	9.4	14307	3.6	5
62	8.3	3430	0.39	1.7	55.6	7.4	23	0.05	150	0	9.4	14307	3.6	5

63	2.1	11800	0.39	5.9	55.6	7.4	23	0.05	150	0	9.4	14307	3.6	5
64	3.1	23600	0.39	11.8	55.6	7.4	23	0.05	150	0	9.4	14307	3.6	5
65	2.9	46360	0.39	23.1	55.6	7.4	23	0.05	150	0	9.4	14307	3.6	5
66	13.5	1380	0.39	0.83	81.4	7.4	23	0.25	150	0	9.4	14307	3.6	5
67	7	3430	0.39	1.7	81.4	7.4	23	0.25	150	0	9.4	14307	3.6	5
68	2.1	11800	0.39	5.9	81.4	7.4	23	0.25	150	0	9.4	14307	3.6	5
69	1.9	23600	0.39	11.8	81.4	7.4	23	0.25	150	0	9.4	14307	3.6	5
70	2	46360	0.39	23.1	81.4	7.4	23	0.25	150	0	9.4	14307	3.6	5
71	12.2	1380	0.39	0.83	102	7.4	23	0.5	150	0	9.4	14307	3.6	5
72	5.8	3430	0.39	1.7	102	7.4	23	0.5	150	0	9.4	14307	3.6	5
73	2	11800	0.39	5.9	102	7.4	23	0.5	150	0	9.4	14307	3.6	5
74	2.9	23600	0.39	11.8	102	7.4	23	0.5	150	0	9.4	14307	3.6	5
75	2	46360	0.39	23.1	102	7.4	23	0.5	150	0	9.4	14307	3.6	5
76	10.2	1380	0.39	0.83	113	7.4	23	1	150	0	9.4	14307	3.6	5
77	6.8	3430	0.39	1.7	113	7.4	23	1	150	0	9.4	14307	3.6	5
78	2.7	11800	0.39	5.9	113	7.4	23	1	150	0	9.4	14307	3.6	5
79	2.9	23600	0.39	11.8	113	7.4	23	1	150	0	9.4	14307	3.6	5
80	2.5	46360	0.39	23.1	113	7.4	23	1	150	0	9.4	14307	3.6	5
81	0.6	29000	0.214	8.05	161	7.4	25	20	150	0	7	66000	−2.4	6
82	1.8	29000	0.122	4.55	161	7.4	25	20	150	0	7	66000	−2.4	6
83	21	29000	0.081	3.03	161	7.4	25	20	150	0	7	66000	−2.4	6
84	39	29000	0.06	2.25	161	7.4	25	20	150	0	7	66000	−2.4	6

85	62	29000	0.049	1.85	161	7.4	25	20	150	0	7	66000	−2.4	6
86	103	29000	0.026	0.96	161	7.4	25	20	150	0	7	66000	−2.4	6
87	114	29000	0.015	0.6	161	7.4	25	20	150	0	7	66000	−2.4	6
88	36.5	32300	0.21	10.9	112	6.4	25	0.29	0	0.12	5.8	44700	0.53	7
89	177	22200	0.18	7.6	112	6.4	25	0.29	0	0.12	5.8	44700	0.53	7
90	404	19000	0.14	6	112	6.4	25	0.29	0	0.12	5.8	44700	0.53	7
91	275	20000	0.05	4	112	6.4	25	0.29	0	0.12	5.8	44700	0.53	7
92	259	20000	0.04	3.8	112	6.4	25	0.29	0	0.12	5.8	44700	0.53	7
93	182	22000	0.02	3.2	112	6.4	25	0.29	0	0.12	5.8	44700	0.53	7
94	50	32300	0.21	10.9	220	7.4	25	0.29	20	0.12	5.8	44700	−0.47	7
95	57	22200	0.18	7.6	220	7.4	25	0.29	20	0.12	5.8	44700	−0.47	7
96	130	19000	0.14	6	220	7.4	25	0.29	20	0.12	5.8	44700	−0.47	7
97	357	20000	0.05	4	220	7.4	25	0.29	20	0.12	5.8	44700	−0.47	7
98	388	20000	0.04	3.8	220	7.4	25	0.29	20	0.12	5.8	44700	−0.47	7
99	323	22000	0.02	3.2	220	7.4	25	0.29	20	0.12	5.8	44700	−0.47	7
100	25	32300	0.21	10.9	85	7.4	25	0.29	70	0.12	5.8	44700	−0.47	7
101	31	22200	0.18	7.6	85	7.4	25	0.29	70	0.12	5.8	44700	−0.47	7
102	31	19000	0.14	6	85	7.4	25	0.29	70	0.12	5.8	44700	−0.47	7
103	187	20000	0.05	4	85	7.4	25	0.29	70	0.12	5.8	44700	−0.47	7
104	223	20000	0.04	3.8	85	7.4	25	0.29	70	0.12	5.8	44700	−0.47	7
105	113	22000	0.02	3.2	85	7.4	25	0.29	70	0.12	5.8	44700	−0.47	7
106	26	32300	0.21	10.9	47	7.4	25	0.29	120	0.12	5.8	44700	−0.47	7

107	36	22200	0.18	7.6	47	7.4	25	0.29	120	0.12	5.8	44700	−0.47	7
108	58	19000	0.14	6	47	7.4	25	0.29	120	0.12	5.8	44700	−0.47	7
109	109	20000	0.05	4	47	7.4	25	0.29	120	0.12	5.8	44700	−0.47	7
110	120	20000	0.04	3.8	47	7.4	25	0.29	120	0.12	5.8	44700	−0.47	7
111	42	22000	0.02	3.2	47	7.4	25	0.29	120	0.12	5.8	44700	−0.47	7
112	195	19000	0.14	6	38	6.4	4	2	0	0	5.8	14307	4.6	7
113	256	20000	0.05	4	38	6.4	4	2	0	0	5.8	14307	4.6	7
114	270	20000	0.04	3.8	38	6.4	4	2	0	0	5.8	14307	4.6	7
115	330	22000	0.02	3.2	38	6.4	4	2	0	0	5.8	14307	4.6	7
116	46.7	20000	0.05	4	40	7.4	4	2	20	0	5.8	14307	3.6	7
117	58	20000	0.04	3.8	40	7.4	4	2	20	0	5.8	14307	3.6	7
118	130	22000	0.02	3.2	40	7.4	4	2	20	0	5.8	14307	3.6	7
119	24	20000	0.05	4	32	7.4	4	2	70	0	5.8	14307	3.6	7
120	30	20000	0.04	3.8	32	7.4	4	2	70	0	5.8	14307	3.6	7
121	57	22000	0.02	3.2	32	7.4	4	2	70	0	5.8	14307	3.6	7
122	8.7	19000	0.14	6	20	7.4	4	2	120	0	5.8	14307	3.6	7
123	5.8	20000	0.05	4	20	7.4	4	2	120	0	5.8	14307	3.6	7
124	8.7	20000	0.04	3.8	20	7.4	4	2	120	0	5.8	14307	3.6	7
125	18	22000	0.02	3.2	20	7.4	4	2	120	0	5.8	14307	3.6	7

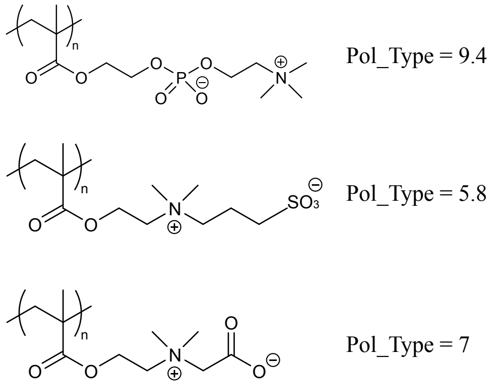

Figure S1. Structures of investigated zwitterionic polymers and numbers of nonfreezing water (Poly_Type value).

Machine Learning using the Full Dataset

In this study, all data sets presented in Table S2 were used for machine learning, particularly using random forest regression model. Entries 1 and 2 exhibited an extremely large value of protein adsorption compared with the rest of the data. This is because the amount of adsorption on the substrate itself is also high. These data points considerably influenced the prediction results, as shown in Figure S1. In Figure S1, R^2 value and RMSE in the test data were 0.50 and 64, respectively, indicating low prediction accuracy. Therefore, further validation was conducted in this study using 123 data points, excluding these two data points.

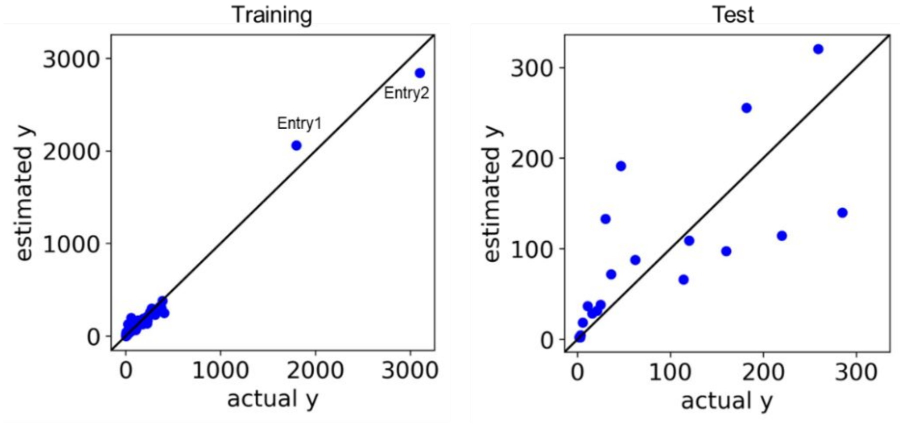

Figure S2. Predicted amounts of protein on ZI-polymer brush using the random forest regression algorithm plotted as a function of corresponding experimental values.

Consideration of polymer configuration

To consider the influence of brush configuration, data samples with s/2R_F > 1 (i.e., data samples under the mushroom condition) were excluded from the dataset, and ML with the RFR model was performed (Figure S3). R_{train}^2 and R_{test}^2 were 0.93 and 0.74, respectively; therefore, no clear improvement in prediction accuracy was observed, indicating that the $s/2R_F$ threshold does not contribute to the improvement of the ML model. Further, the outlier data samples in Figure 5(iii) were all obtained from the same literature.[7] Therefore, it is assumed that the error in Figure 5(iii) depends on the experimental environment of the respective literature.

.

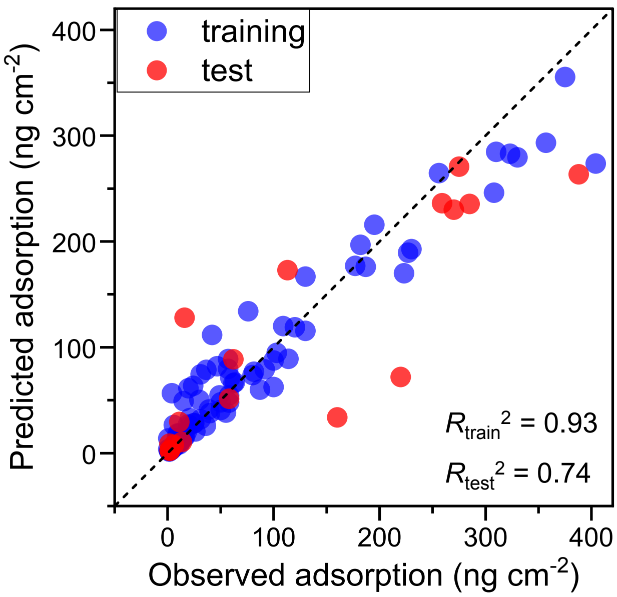

Figure S3. Prediction performance of machine learning using a dataset excluding data samples with s/2R_F > 1.

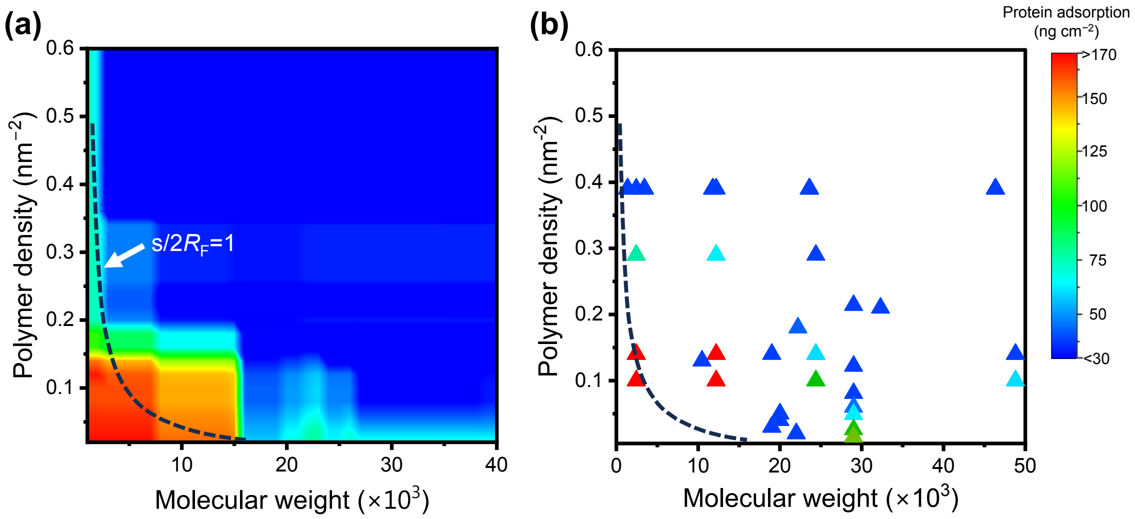

Figure S4. Color map of (a) predicted and (b) experimental protein adsorption with a boundary of $s/2R_F = 1$. The degree of protein adsorption is predicted using a trained RFR model, and the descriptors Ionic Strength, Sub_Ad, Pro_Conc, and Flow Rate were fixed as in Figure 6.
